# IMP3, CDK4, MDM2 and β-catenin expression in Enchondroma and Central Chondrosarcoma: Diagnostic and prognostic utility

**DOI:** 10.1016/j.clinsp.2024.100483

**Published:** 2024-10-05

**Authors:** Daniele Moraes Losada, Maurício Etchebehere, Francisco Fontes Cintra, Eliane Maria Ingrid Amstalden

**Affiliations:** a Department of Pathology, Universidade Estatual de Campinas (Unicamp), Campinas, SP, Brazil; b Hospital de Clínicas da Universidade Estadual de Campinas (Unicamp), Campinas, SP, Brazil

**Keywords:** Chondroma, Chondrosarcoma, Cyclic IMP, Cyclin-dependent kinase 4, Proto-oncogene proteins c-mdm2, Beta catenin, Immunohistochemistry

## Abstract

**Introduction:**

The role of IMP3, CDK4, MDM2 and β-catenin proteins in Enchondroma and Central Chondrosarcoma is not totally understood. The aim of this study is to evaluate the immunoexpression of these proteins, associating histological grade, clinical data and prognosis to these tumors.

**Methods:**

This is a retrospective-analytical study of 32 Enchondroma and 70 Central Chondrosarcoma.

**Results:**

IMP3, CDK4, MDM2 and β-catenin expression was observed in 22.82 %, 13.82 %, 17.17 % and in 8.8 % of cases, respectively. All Enchondromas positive for these immunomarkers were located in short tubular bones. The positivity for these antibodies is directly proportional to Chondrosarcoma's histological grade increase. No difference was found between Enchondroma and Chondrosarcoma, Grade 1 for IMP3, CDK4 and ß-catenin positivity. Significant metastasis outcome was observed for IMP3, CDK4, MDM2 and death for MDM2 expression.

**Conclusion:**

IMP3, CDK4, MDM2 and β-catenin expression in Enchondromas of short bones phenotypically characterizes these tumors. Their expression has not proven to be useful either as diagnostic markers of these neoplasms or in distinguishing between Enchondroma and Chondrosarcoma, Grade 1. The significant immunoexpression of IMP3, CDK4 and MDM2 in metastatic Chondrosarcoma and the lower survival in those with positivity for MDM2 suggest a possible association of these proteins with tumor aggressiveness.

## Highlights

• IMP3, CDK4, MDM2 and β-catenin are expressed in Enchondromas of short bones.
• These antibodies are useless in distinguishing Enchondroma and Chondrosarcoma G1.
• Their positivity is proportional to Chondrosarcomas histological grade increase.
• The positivity of IMP3, CDK4 and MDM2 is associated to metastasis.
• The expression of MDM2 is associated with a worse prognosis related to death.

## Introduction

Enchondroma (ENC) ranks second in frequency, accounting for approximately 10 % to 25 % of all benign bone neoplasms.^1^^,^^2^ Central Chondrosarcoma (CC) has the second highest incidence among primary malignant bone tumors and histologically is graded into: Low-Grade (Atypical Cartilaginous Tumor/Chondrosarcoma, Grade 1) (LGC), Intermediate-Grade (Chondrosarcoma, Grade 2) (IGC) and High-Grade (Chondrosarcoma, Grade 3) (HGC), according to WHO (2020).^1^^,^^2^ The frequency of adverse events (recurrence, metastasis and death) is directly related to tumor progression in this group of neoplasms; therefore, HGC is the most aggressive and prone to metastases.^2^^,^^3^

Distinguishing between ENC and LGC can be difficult and is subject to great interobserver variation, which requires joint evaluation with clinical and radiological data.^4^ These are histologically similar nosological entities but with different biological behavior. The fundamental difference between an ENC and LGC translates into the limited growth potential of the ENC compared to the slow but continuous and locally invasive growth pattern of LGC.^1^^,^^2^ The clinical course of an LGC is dependent on localization and the possibility of surgical removal.^1^

Research groups are looking for complementary diagnostic tools that could improve the understanding of the pathogenesis of cartilaginous neoplasms, resulting in greater diagnostic accuracy and therapeutic advances.^5^, ^6^, ^7^, ^8^, ^9^, ^10^, ^11^

Several oncogenic signaling pathways have been implicated in the progression of cartilaginous tumors, such as those related to cell migration (IMP3) and cell cycle (CDK4, MDM2, and β-catenin), which should be better elucidated. IMP3 is a member of the oncofetal protein family and plays an important role in mRNA trafficking and stabilization, cell growth and migration during the early stages of embryogenesis.^12^ In addition, it contributes to the development of cancer, through the formation of cellular structures similar to podosomes. These structures are related to the extension of the extracellular matrix and the destruction of the surrounding matrix, increasing the invasive capacity of malignant cells.^13^, ^14^, ^15^ Shooshtarizadeh et al.^5^ observed that IMP3 overexpression correlates with high histological grade in Chondrosarcomas acting as a possible facilitator of tumor progression.

CDK4 is a protein involved in the cell cycle that regulates cell transit at the G1 restriction point through hyperphosphorylation of the pRb pathway.^6^ Promotion of the pRb pathway related to cell cycle control through CDK4 amplification is observed in many tumors.^6^ In addition to CDK4, the 12q13 gene region harbors the MDM2 gene that is frequently co-amplified with CDK4.^16^ MDM2 is a protein involved in the cell cycle through the p53 pathway.^16^ The MDM2 gene encodes an E3 ubiquitin ligase involved in the degradation of the p53 tumor suppressor protein involved in cell cycle arrest and/or induction of apoptosis.^16^ Schrage et al. ^6^ demonstrated that alterations in the pRb pathway and p53 pathway are important in the tumor progression of cartilaginous neoplasms.

Β-catenin is a protein involved in the cell cycle that activates canonical Wnt signaling pathways.^17^ The Wnt/β-catenin pathway is a highly complex and unique signaling pathway, contributing to the regulation of several functions related to cell proliferation, migration, renewal and regeneration during tissue homeostasis, as well as those related to organogenesis during embryonic development.^18^ When altered, it interferes with both the regulation of gene expression and cell invasion, migration, proliferation and differentiation; facilitating metastatic events.^18^, ^19^, ^20^, ^21^, ^22^ In the group of cartilaginous mesenchymal neoplasms, Schrage et al. ^23^ observed that activation of the canonical Wnt signaling pathway may play an important role in the transition from benign to malignant central cartilaginous lesions; however, it is not crucial for tumor progression.

The role of IMP3, CDK4, MDM2 and β-catenin proteins in Enchondroma and Central Chondrosarcoma is not totally understood. In this study, the authors aimed to evaluate the immunoexpression of these proteins, associating histological grade, clinical data and prognosis to these tumors.

## Methodology

This is a retrospective-analytical study. Data collection took place after approval by the institutional Research Ethics Committee (CAAE: 02184318.6.0000.5404). One hundred and two patients (37 men and 65 women, aged 9 to 87), diagnosed with ENC or CC by biopsy and/or resection specimens, were identified from the Clinical Hospital, at the State University of Campinas, from 1994 to 2019. The tumor specimens were routinely fixed in 10.00 % formalin and later decalcified with hydrochloric acid and ethylenediaminetetraacetic acid. Atypical Cartilaginous Tumor and Chondrosarcoma, Grade 1, due to histological and prognostic similarities, were grouped in Low Grade Chondrosarcoma (LGC) category for statistical purposes. The samples consisted of 32 cases of ENC and 70 cases of CC, including 29 LGC, 33 IGC and 8 HGC. Clinical data (age, gender, location and type of bone affected) and outcomes were obtained by reviewing patients’ medical records. It was considered a favorable outcome in the absence of adverse events (death, recurrence or metastasis) or unfavorable if at least one adverse event was present. Representative formalin-fixed, paraffin-embedded blocks were selected for the immunohistochemistry study. Of the 102 cases, 92 cases were tested for the IMP3 antibody, 94 cases for the CDK4 antibody, 99 cases for the MDM2 antibody and 90 cases for the β-catenin antibody. The loss of some analyses was due to technical difficulties.

### Immunohistochemical technique and analysis

**Fig. 1:**
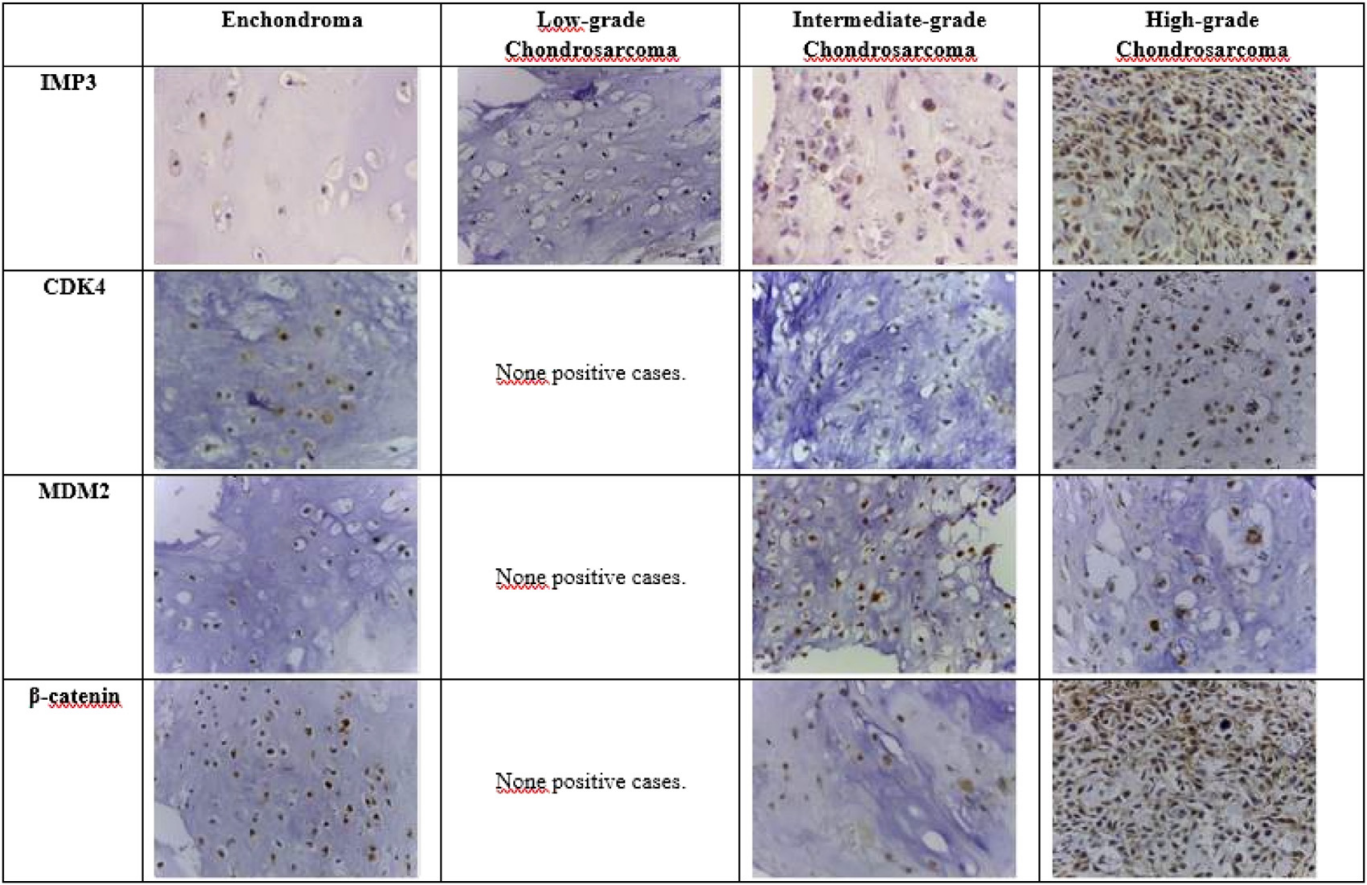
Photomicrographs (original magnification 400×) of Enchondroma and Central Chondrosarcoma with positive immunoexpression for IMP3 (cytoplasmic labeling) and for CDK4, MDM2 and β-catenin (nuclear labeling).

The primary antibodies which were used included anti-IMP3, clone EP286 (1/100 dilution, Cell Marque), anti-CDK4, clone EP180 (1/100 dilution, Cell Marque), anti-MDM2, clone IF2 (1/100 dilution, ZETA) and anti-β-catenina, clone EP286 (1/100 dilution, ZETA). Immunohistochemical staining was performed on 5 µm-thick sections processed from formalin-fixed paraffin-embedded tissues, which were mounted on silanized slides and were briefly deparaffinized in xylol and rehydrated in serial alcohol. Epitope retrieval was achieved by steaming with citrate buffer (at 95 °C). The EnVision + Dual Link System HRP polymer (Dako) was used as a reaction amplifier. The antibody complex was visualized with 3.3′-diaminobenzidine tetrahydrochloride (Dako). The sections were counterstained with hematoxylin. The appropriate negative and positive controls were included in each assay as per the manufacturer's instructions. Assessment of immunohistochemical staining was evaluated by two independent pathologists (DML and EMIA) who were blinded to the clinicopathological parameters of the patients. The IMP3-positive tumor cell showed immunoreactivity in cytoplasm while CDK4, MDM2 and β-catenin positive tumor cells showed nuclear staining, regardless of the intensity or quantity of immunostained cells (Fig. 1). The count of positive and negative cells was performed using digital photographs, obtained through a photomicroscope (LEICA ICC50 HD) and computed with the aid of a digital camera (Tucsen ISH-500®). A minimum of 5 fields of higher magnification (40× objective) were photographed, in the areas of greatest expression (“hot spots”) previously identified with a 10× objective. A minimum of 100 cells were analyzed for each case. Positive and negative cells were computed with the aid of specific software (Image J®).

### Statistical analysis

The data analysis was performed using the SAS System for Windows version 9.4 (SAS Institute Inc., Cary, NC, USA). Chi-Square and Fisher's exact tests were used to compare categorical variables. To compare numeric variables, the Mann-Whitney and Kruskal-Wallis tests were performed, followed by Dunn's posthoc test when necessary. Survival analyses were performed considering disease-specific survival, which was defined as the time from diagnosis until the presence of death outcome or last follow-up. Univariate Cox regressions were also performed. All variables with a p-value <0.10 on univariate Cox regressions were included in a multivariate model with a stepwise selection method, in order to identify independent risk factors associated with survival, *p* < 0.05 was considered statistically significant.

## Results

The results are summarized in Table 1, Table 2, Table 3, Table 4, Table 5, Table 6, Table 7.

**Table 1:** Descriptive analysis and comparisons between diagnostic groups.

Parameters		Enchondroma	Low-grade Chondrosarcoma	Intermediate-grade Chondrosarcoma	High-grade Chondrosarcoma	p-value
**N**		32	29	33	8	
**Follow-up**	Mean ± DP	37.9 ± 40.3	49.8 ± 47.8	62.9 ± 42.8	39.8 ± 31.9	**0.0490**^a^^,^^c^
	Median (min‒max)	24.0 (5.0‒130.0)	29.0 (4.0‒228.0)	70.0 (8.0‒168.0)	33.0 (7.0‒110.0)	
**Age**	Mean ± DP	31.3 ± 15.3	46.7 ± 17.0	52.4 ± 14.6	47.9 ± 16.0	**<0.0001**^a^^,^^b^
	Median (min‒max)	28.0 (9.0‒61.0)	47.0 (18.0‒83.0)	54.0 (22.0‒87.0)	48.5 (24.0‒67.0)	
**Gender**	Male	11 (34.4 %)	10 (34.5 %)	11 (33.3 %)	5 (62.5 %)	0.4585^d^
	Female	21 (65.6 %)	19 (65.5 %)	22 (66.7 %)	3 (37.5 %)	
**Topography**	Short bone	26 (81.3 %)	0 (0.0 %)	0 (0.0 %)	1 (12.5 %)	**<0.0001**^e^
	Long bone	6 (18.8 %)	24 (82.8 %)	20 (60.6 %)	4 (50.0 %)	
	Flat bone	0 (0.0 %)	5 (17.2 %)	13 (39.4 %)	3 (37.5 %)	
**Prognosis**	Favorable	32 (100.0 %)	26 (89.7 %)	22 (66.7 %)	5 (62.5 %)	**0.0002**^e^
	Unfavorable	0 (0.0 %)	3 (10.3 %)	11 (33.3 %)	3 (37.5 %)	
**Recurrence**	No	32 (100.0 %)	28 (96.6 %)	28 (84.8 %)	7 (87.5 %)	**0.0475**^e^
	Yes	0 (0.0 %)	1 (3.4 %)	5 (15.2 %)	1 (12.5 %)	
**Metastasis**	No	32 (100.0 %)	28 (96.6 %)	30 (90.9 %)	6 (75.0 %)	**0.0300**^e^
	Yes	0 (0.0 %)	1 (3.4 %)	3 (9.1 %)	2 (25.0 %)	
**Death**	No	32 (100.0 %)	28 (96.6 %)	27 (81.8 %)	6 (75.0 %)	**0.0079**^e^
	Yes	0 (0.0 %)	1 (3.4 %)	6 (18.2 %)	2 (25.0 %)	

*Follow-up time in months.

a Based on Kruskal-Wallis test.

b Dunn's test: differences between 1 and 2, 3, 4.

c Difference between 1 and 3.

d Based on Chi-Square's test.

e Based on Fisher's Exact test.

The follow-up time for patients with ENC ranged between 5 and 130 months (mean 37.94 months) and for CC ranged from 4 to 228 months (mean 54.79 months). There was a significant difference between the age at diagnosis of patients with ENC (ranging from 9 to 61 years, mean 31.25 years) compared to those with CC (ranging from 18 to 87 years, mean 49.50 years) with *p* < 0.0001. No differences were found between gender and diagnosis (*p* = 0.4585). Twenty-six cases of ENC were located at short bones of the extremities, six cases in long bones, and none in flat bones. The LGC group totaled 29 cases, 24 of which were atypical cartilaginous tumors, all located in the long bone, and 5 Chondrosarcomas, Grade 1, 4 being distributed in the costal arch and 1 in the iliac. IGC was observed predominantly in long bones (60.6 %), as well as HGC (50.0 %) of the cases. The topography of ENC was preferentially in short tubular bones (81.3 % of cases), while CC was located in long tubular bones (68.6 % of cases), with statistical significance (*p* < 0.0001) (Table 1).

A total of seventeen patients had an unfavorable outcome: 3 out of 29 (10.3 %) in the LGC group (1 recurrence; 1 metastasis and 1 death); 11 out of 33 (33.3 %) in the IGC (03 recurrences; 1 metastasis; 4 deaths; 1 recurrence plus metastasis; 1 recurrence plus death and 1 metastasis plus death) and 3 out of 8 (37.5 %) in HGC (1 death; 1 recurrence plus metastasis and 1 metastasis plus death). There was significance between the groups regarding unfavorable outcomes (*p* = 0.0002) and adverse events (recurrence with *p* = 0.0475; metastasis with *p* = 0.0300; death with *p* = 0.0079). None of the patients diagnosed with Enchondroma showed adverse events (*p* = 0.0023) (Table 1). The logistic regression analysis of factors related to unfavorable outcomes confirmed the location of the lesion as a predictive factor, with the involvement of flat bones having a worse prognosis when compared to tumors of long and short bones (*p* = 0.0435) (Table 2).

**Table 2:** Cox logistic regression for clinical factors associated with overall survival.

Univariate analysis				
Parameters	Specification	p-value	HR	95 % CI
**Age**		0.0684	1.036	0.997; 1.077
**Gender**	Male vs. Female	0.8177	0.849	0.210; 3.429
**Topography**	Flat bones vs. (Short bones + Long bones)	**0.0026**	11.389	2.339; 55.459
**Recurrence**	Yes vs. No	0.9227	0.900	0.108; 7.529
**Metastasis**	Yes vs. No	**0.0130**	8.620	1.576; 47.144
**Multivariate analysis**				
**Parameters**	**Specification**	**p-value**	**HR**	**95 % CI**
**Topography**	Flat bones vs. (Short + Long bones)	**0.0435**	6.111	1.055; 35.411

### Immunohistochemical analysis

**Fig. 2:**
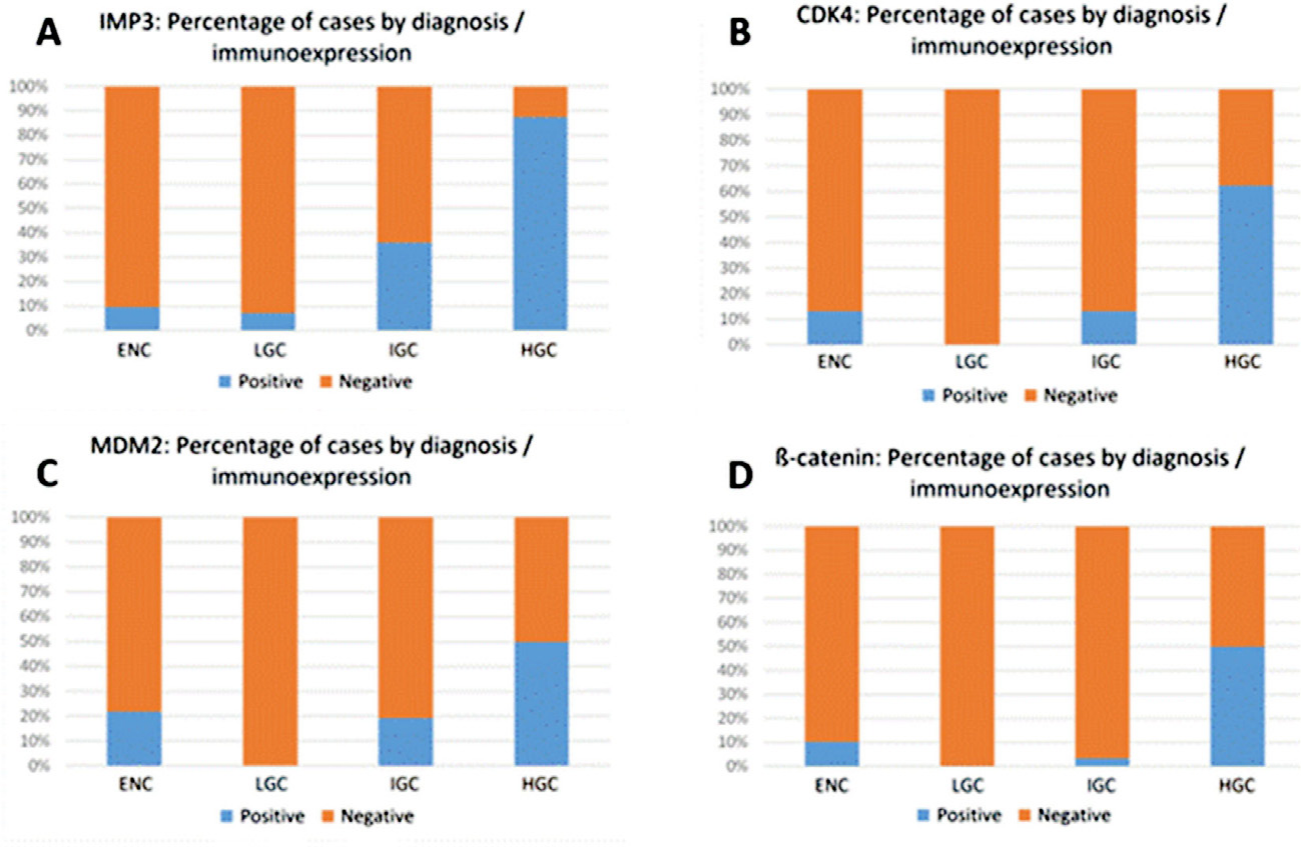
Percentage of cases with immunoexpression in Enchondroma (ENC) and Central Chondrosarcoma of Low-Grade (LGC), Intermediate-Grade (IGC) and High-Grade (HGC). Fisher's exact test. (A) ENC vs. LGC (*p* = 1.0000); LGC vs. IGC (*p* = 0.0160); LGC vs. HGC (*p* = 0.0001) and IGC vs. HGC (*p* = 0.0167). (B) ENC vs. LGC (*p* = 0.1153); LGC vs. IGC (*p* = 0.1153); LGC vs. HGC (*p* = 0.0002) and IGC vs. HGC (*p* = 0.0101). (C) ENC vs. LGC (*p* = 0.0118); LGC vs. IGC (*p* = 0.0247); LGC vs. HGC (*p* = 0.0012) and IGC vs. HGC (*p* = 0.1672). (D) ENC vs. LGC (*p* = 0.2424); LGC vs. IGC (*p* = 1.0000); LGC vs. HGC (*p* = 0.0019) and IGC vs. HGC (*p* = 0.0048).

**Fig. 3:**
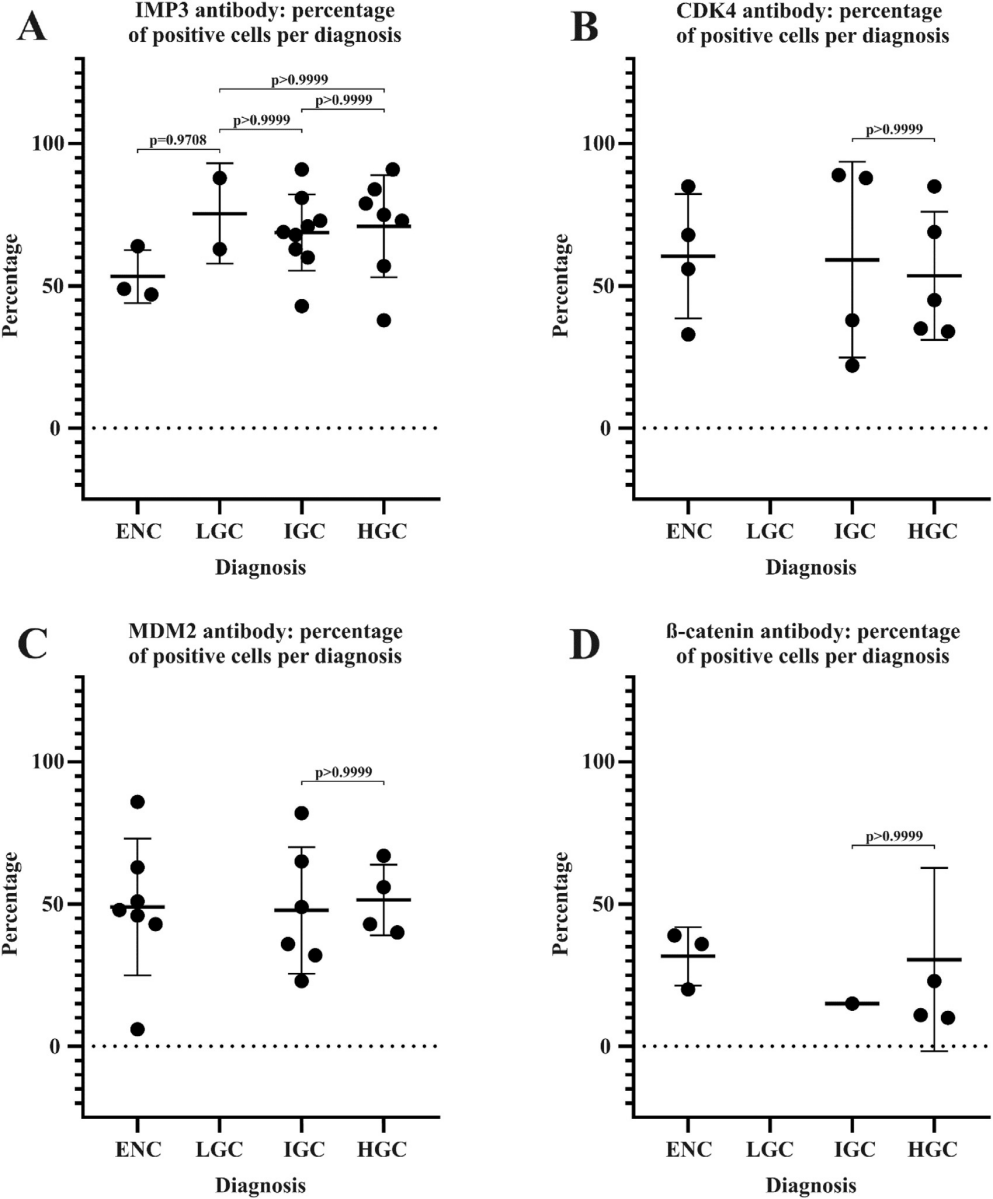
Percentage of positive cells for IMP3, CDK4, MDM2 and β-catenin in Enchondroma (ENC) and Central Chondrosarcoma of Low-Grade (LGC), Intermediate-Grade (IGC) and High-Grade (HGC). Kruskal Wallis test followed by Dunn's test. (A) ENC vs. LGC (*p* = 0.9708); LGC vs. IGC (*p* > 0.9999); LGC vs. HGC (*p* > 0.9999) and IGC vs. HGC (*p* > 0.9999). (B) IGC vs. HGC (*p* > 0.9999). (C) IGC vs. HGC (*p* > 0.9999). (D) IGC vs. HGC (*p* > 0.9999).

The immunoexpression of IMP3 was observed in 21 of 92 (22.82 %) cases, CDK4 in 13 of 94 (13.82 %) cases, MDM2 in 17 of 99 (17.17 %) cases, and β-catenin in 8 of 90 (8.8 %) cases (Fig. 2), with no significance for percentage of positive cells between the analyzed tumor's group (Fig. 3).

IMP3 positive cases were distributed as follows: 3 of the 31 ENC (9.7 %), 2 of the 28 LGC (7.1 %), 9 of the 25 IGC (36 %) and 7 of the 8 HGC (87.5 %). A significantly higher IMP3 expression was observed in CC when compared to ENC: 18 of the 61 CC (29.5 %) and 3 of the 31 ENC (9.7 %) (*p* = 0.0322). Comparison between groups revealed: ENC and LGC (*p* = 1.0000), LGC and IGC (*p* = 0.0160), LGC and HGC (*p* = 0.0001) and IGC and HGC (*p* = 0.0167) (Fig. 2A). The 21 positive cases showed the following distribution: 4 in short bones (3 ENC and 1 HGC), 11 in long bones (2 LGC, 6 IGC and 3 HGC) and 6 in flat bones (3 IGC and 3 HGC). Unfavorable outcomes occurred in 4 of the 18 CC-positive cases (22.2 %): 1 with metastasis, 2 with recurrence plus metastasis, and 1 with metastasis plus death. IMP3 expression was significant in patients that evolved with metastasis (*p* = 0.0236) (Table 3).

**Table 3:** Fisher's Exact Test for IMP3 antibody expression and adverse events in patients diagnosed with Central Chondrosarcoma (*n* = 61).

Parameters		IMP3 Positive	IMP3 Negative	p-value
**N**		18	43	
**Recurrence**	Yes	2 (11.1 %)	3 (7 %)	0.6266
	No	16 (88.9 %)	40 (93 %)	
**Metastasis**	Yes	4 (22.2 %)	1 (2.3 %)	**0.0236**
	No	14 (77.8 %)	42 (97.7 %)	
**Death**	Yes	1 (5.6 %)	7 (16.3 %)	0.4165
	No	17 (94.4 %)	36 (83.7 %)	

Immunoreactivity for CDK4 was observed as follows: 4 of the 30 ENC (13.3 %), 4 of the 30 IGC (13.3 %) and 5 of the 8 HGC (62.5 %). None of the 26 LGC were positive for this antibody. There was no significance comparing ENC and CC-positive cases (*p* = 1.0000). Comparison between groups revealed ENC and LGC (*p* = 0.1153), LGC and IGC (*p* = 0.1153), LGC and HGC (*p* = 0.0002), and IGC and HGC (*p* = 0.0101) (Fig. 2B). The 13 positive cases showed the following distribution: 5 at short bones (3 ENC and 1 HGC), 3 in long bones (2 IGC and 1 HGC) and 5 in flat bones (3 IGC and 3 HGC). The unfavorable outcome was observed in 4 of the 9 CC positive cases (44.4 %): 1 with metastasis, 1 with death, 1 with recurrence plus metastasis and 1 with metastasis plus death. CDK4 expression was significant in patients that evolved with metastasis (*p* = 0.0320) (Table 4).

**Table 4:** Fisher's Exact Test for CDK4 antibody expression and adverse events in patients diagnosed with Central Chondrosarcoma (*n* = 64).

Parameters		CDK4 Positive	CDK4 Negative	p-value
**N**		9	55	
**Recurrence**	Yes	1 (11.1 %)	5 (9.1 %)	1
	No	8 (88.9 %)	50 (90.9 %)	
**Metastasis**	Yes	3 (33.3 %)	3 (5.5 %)	**0.0320**
	No	6 (66.7 %)	52 (94.5 %)	
**Death**	Yes	2 (22.2 %)	7 (12.7 %)	0.6021
	No	7 (77.8 %)	48 (87.3 %)	

The expression of MDM2 antibody revealed the following values: 7 of the 32 ENC (21.9 %), 6 of the 31 IGC (19.4 %), and 4 of the 8 HGC (50.0 %). None of the 28 LGC were positive for this antibody. There was no significance comparing ENC and CC-positive cases (*p* = 0.3911). Comparison between groups revealed: ENC and LGC (*p* = 0.0118), LGC and IGC (*p* = 0.0247), LGC and HGC (*p* = 0.0012) and IGC and HGC (*p* = 0.1672) (Fig. 2C). The 17 positive cases showed the following distribution: 8 in short bones (7 ENC and 1 HGC), 3 in long bones (2 IGC and 1 HGC) and 6 in flat bones (4 IGC and 2 HGC). Unfavorable outcomes occurred in 5 of the 10 CC-positive cases (50.0 %): 2 with death; 1 with recurrence plus metastasis and 2 with metastasis plus death. MDM2 expression was significant in patients who evolved with metastasis (*p* = 0.0387) and death (*p* = 0.0139) (Table 5). Logistic regression evidenced that MDM2 expression was associated with death (*p* = 0.0435) (Table 6).

**Table 5:** Fisher's Exact Test for MDM2 antibody expression and adverse events in patients diagnosed with Central Chondrosarcoma (*n* = 67).

Parameters		MDM2 Positive	MDM2 Negative	p-value
**N**		10	57	
**Recurrence**	Yes	1 (10 %)	6 (10.5 %)	1
	No	9 (90 %)	51 (89.5 %)	
**Metastasis**	Yes	3 (30 %)	3 (5.3 %)	**0.0387**
	No	7 (70 %)	54 (94.7 %)	
**Death**	Yes	4 (40 %)	4 (7 %)	**0.0139**
	No	6 (60 %)	53 (93 %)

**Table 6:** Cox logistic regressions for MDM2 imunoexpression and death.

Univariate analysis				
Parameter	Specification	p-value	HR	95 % CI
**MDM2**	Positive vs. Negative	**0.0047**	8.732	1.946; 39.186
**Multivariate analysis**				
**Parameter**	**Specification**	**p-value**	**HR**	**95 % CI**
**MDM2**	Positive vs. Negative	**0.0493**	6.157	1.006; 37.689

The values detected for β-catenin antibodies were: 3 of the 29 ENC (10.35 %), 1 of the 29 IGC (3.45 %), and 4 of the 8 HGC (50.00 %). None of the 24 LGC were positive for this antibody. There was no significance comparing ENC and CC-positive cases (*p* = 0.7092). Comparison between groups revealed: ENC and LGC (*p* = 0.2424); LGC and IGC (*p* = 1.0000); LGC and HGC (*p* = 0.0019) and IGC and HGC (*p* = 0.0048) (Fig. 2D). The 8 positive cases showed the following distribution: 4 in short bones (3 ENC and 1 HGC), 1 in long bones (1 HGC) and 3 in flat bones (1 IGC and 2 HGC). An unfavorable outcome was observed in 1 of the 5 CC positive cases (20.0 %) (Table 7).

**Table 7:** Fisher's Exact Test for β-catenin antibody expression and adverse events in patients diagnosed with Central Chondrosarcoma (*n* = 61).

Parameters		β-catenina Positive	β-catenina Negative	p-value
**N**		5	56	
**Recurrence**	Yes	0 (0 %)	5 (8.9 %)	1
	No	5 (100 %)	51 (91.1 %)	
**Metastasis**	Yes	1 (20 %)	4 (7.1 %)	0.3579
	No	4 (80 %)	52 (92.9 %)	
**Death**	Yes	1 (20 %)	6 (10.7 %)	0.4684
	No	4 (80 %)	50 (89.3 %)	

Concomitant immunoexpression was evaluated as follows: 90 cases for CDK4 and MDM2, 84 cases for CDK4 and β-catenin, 89 for MDM2 and β-catenin and 83 for CDK4, MDM2 and β-catenin. There was co-expression of CDK4 and MDM2 in 3 of 30 ENC (10.0 %), 9 of 25 LGC (0 %), 2 of 27 IGC (7.4 %) and 4 of 8 HGC (50.0 %), with significance when comparing groups LGC and HGC and IGC and HGC (*p* = 0.0019). There was co-expression of CDK4 and β-catenin in 1 of 28 ENC (3.6 %), 9 of 22 LGC (0 %), 9 of 26 IGC (0 %) and 4 of 8 HGC (50.0 %), with significance when comparing groups ENC and HGC, LGC and HGC and IGC and HGC (*p* = 0.0002). There was co-expression of MDM2 and β-catenin in 2 of 29 ENC (6.9 %), 9 of 24 LGC (0 %), 9 of 28 IGC (0 %) and 3 of 8 HGC (37.5 %), with significance when comparing groups ENC and HGC, LGC and HGC and IGC and HGC (*p* = 0.0016). There was co-expression of CDK4, MDM2 and β-catenin in 1 of 28 ENC (3.6 %), 9 of 22 LGC (0 %), 9 of 25 IGC (0 %) and 3 of 8 HGC (37.5 %), with significance when comparing groups ENC and HGC, LGC and HGC and IGC and HGC (*p* = 0.0023). The unique ENC case with co-expression of CDK4, MDM2 and β-catenin was located in the short bone of the extremity, in a 31-year-old patient with a favorable outcome. The three HGC cases with co-expression of CDK4, MDM2 and β-catenin exhibited the following clinical characteristics: a 24-year-old patient with a tumor located in a short bone and with a favorable outcome, a 63-year-old patient with a tumor located in a long bone and with favorable outcome and a 52-year-old patient with a tumor located in a flat bone and with unfavorable outcome (metastasis and death).

## Discussion

ENC and CC are cartilaginous matrix-producing tumors with distinct biological behavior and management ranging from radiological follow-up to radical surgery.^24^ The surgical procedure, when indicated, is currently the treatment of choice, with limited response of these tumors to radio and/or chemotherapy treatments.^25^^,^^26^ Furthermore, the histological and radiological similarity between ENC and LGC may make their distinction a challenge with large interobserver variation.^27^

The clinical findings in this study were similar to those found in the literature^[^^1^^,^^28^ and summarized as follows: lower mean age observed in the ENC group when compared to CC without gender differences, a higher frequency of ENC in short bones of the extremities and of CCs in long bones and flat bones, and worse prognosis in CC located at flat bones compared to tumors of long and short bones. The histological grade of CC showed significant differences in relation to the unfavorable general evolution as well as when individualized by event (recurrence, metastasis and death) and was directly proportional to the increase of the histological grade in accordance with the literature.^1^^,^^2^

Of the three cases of LGC that presented unfavorable progression, one was located in the appendicular skeleton (femur), which evolved with local recurrence, and two in the axial skeleton (scapula and costal arch) which developed metastasis and death, respectively. A possible explanation for the recurrence of the LGC located in the appendicular skeleton is the surgical procedure applied (curettage), since this may leave some tumor cells in loco, which then may continue their growth, configuring a residual lesion and not a true recurrence. The unfavorable evolution observed in the other two cases of LGC which developed either metastasis or death, both located in the axial skeleton (scapula and costal arch), is justified by the already well-documented unfavorable evolution of Chondrosarcomas, even those of low grade, which originate in flat bones.^1^

Several oncogenic signaling pathways have been implicated in the biology of cartilaginous neoplasms and in progressions such as those related to cell migration (IMP3) and cell cycle (CDK4, MDM2, and β-catenin) .^5^^,^^6^^,^^18^^,^^29^, ^30^, ^31^, ^32^, ^33^ In this study, the expression of these proteins, both isolated and grouped, was analyzed in ENC and compared with CC of different histological grades.

Both ENCs and CCs demonstrated IMP3 immunoreactivity, with a higher frequency of expression in the CC group, which was directly proportional to the increase in the histological grade and, therefore, correlated with tumor progression. Similar to this study, Shooshtarizadeh et al.^5^ observed that IMP3 expression correlates with high histological grade in CC and also did not observe differences between groups for the percentage of positive cells. This antibody did not prove useful in distinguishing between ENC and LGC. It is significant that all cases of ENC evaluated in the present study with IMP3 expression were located in the short tubular bones, which may particularize ENCs located in this topography. Similarly, the research group observed the expression of Amphiregulin, a protein also involved in cell migration, exclusively in ENCs located in short tubular bones.^34^ Only one research group has analyzed this antibody in Enchondromas and Chondrosarcomas to date, which did not find positive expression of IMP3 in ENC and, therefore, considered this immunomarker efficient in differentiating between these groups.^5^

A higher frequency of metastasis was observed in patients with positivity for IMP3, which can be explained by its role in carcinogenesis through the formation of cellular structures similar to podosomes, which in turn are related to the extension of the extracellular matrix and destruction of the surrounding matrix, increasing the invasiveness capacity of neoplastic cells.^13^, ^14^, ^15^ Since no invasive capacity is observed in ENCs, future studies should analyze the role of this antibody in the carcinogenesis of this group of lesions, including the correlation of IMP3 immunoexpression with cytoarchitectural and imaging aspects of ENCs located in short bones. One hypothesis to be considered is that this protein exerts a function similar to that described in embryogenesis, promoting cell growth in this group of lesions.

There was CDK4 positive immunoexpression in both ENC and CCs, although more frequently in the latter, and was directly proportional to its increase in histological grade. Interestingly, no positive expression of CDK4 was observed in LGC. A study developed by Schrage et al.^6^ documented increasing positivity of this antibody as related to the histological grade increase in CCs, including positivity in the LGC. The research carried out by Si and Liu^[^^29^ did not demonstrate variation in CDK4 expression related to the histological grade of CC located in the mandible. These results together raise the possibility that the location of the lesion interferes with the CCs signaling pathways. This study documented CDK4 expression in CCs located in short, long and flat bones, with a predominance in lesions located in flat bones. CDK4 amplification stimulates the pRb signaling pathway, which is related to the cell cycle,^35^ and may contribute, at least in part, to tumor growth in these groups of neoplasms. There was no difference between groups for the parameter percentage of positive cells. The percentage of CDK4-positive cells in ENCs and CCs had not been previously described in the literature.

A higher frequency of metastasis was observed in patients with positivity for CDK4 without association with local recurrence. Ouyang et al.^30^ demonstrated an increase in CDK4 expression in patients diagnosed with CC which evolved with metastasis and local recurrence. In addition to its important role in the cell cycle, CDK4 exerts immunomodulatory and immunogenic effects by negatively interfering in the expression of PD-L1 in tumor cells and also in the direct reduction of infiltrating T lymphocytes of the immune system.^35^ Together, these effects may help tumor cells to “bypass” the body's defense system, favoring metastatic events.

The MDM2 antibody showed significant immunoreactivity in both ENC and CC and was directly proportional to the histological grade increase of the latter. No MDM2 expression was observed in the LGC group. There was no difference between groups for the parameter percentage of positive cells. Schrage et al. ^6^ did not detect MDM2 positivity in Enchondromas, but observed expression of this immunomarker in Chondrosarcomas, Grade 1 in addition to its association with tumor progression in this group of lesions. On the other hand, Daugaard et al.^31^ did not show a correlation between MDM2 expression and histological grade in Chondrosarcomas.

The present study documented a higher frequency of metastasis and death related to positivity for MDM2 and no correlation of this antibody with local recurrence. Expression of MDM2 was validated as an independent variable of worse prognosis in Chondrosarcomas, being associated with death. According to Oshiro et al.^32^ changes in the p53 pathway, a tumor suppressor protein degraded by MDM2, are positively correlated with adverse events of recurrence, metastasis and death in patients with chondrosarcomas. The positivity for MDM2 in Enchondroma, documented for the first time in the present study, may be related to the inhibition of the p53 pathway in this group of neoplasms promoting lesion growth, but without necessarily any prognostic implications. Given the important role of the p53 pathway not only in cell survival but also in the mechanisms of cell adhesion, motility and invasion,^36^ inhibition of this pathway can stimulate an array of metastatic facilitators, justifying the higher frequency of metastasis in patients with diagnosis of Chondrosarcoma and positivity for MDM2.

The β-catenin antibody showed positive immunoexpression in both ENC and CC and was directly proportional to later increases in the histological grade. β-catenin expression was not observed in the LGC group. β-catenin positivity correlated with increased histological grade in Chondrosarcomas in recent research by Khademian et al.^18^ Chen et al.^33^ demonstrated nuclear positivity for β-catenin in patients with Enchondroma and Chondrosarcoma with positivity directly proportional to the increase in the histological grade. There was no difference between groups for the parameter percentage of positive cells. The percentage of positive cells for β-catenin was not a parameter evaluated by previous research groups.^18^^,^^33^

There was no association of this antibody with the adverse events of local recurrence, metastasis, or death. Khademian et al.^18^ observed a correlation between β-catenin expression and metastasis in patients diagnosed with Chondrosarcoma. Chen et al.^33^ demonstrated that the survival rate of patients with a diagnosis of Chondrosarcoma and β-catenin positivity was significantly lower than in patients with no β-catenin expression, although this parameter has not proved to be an independent factor of worse prognoses.

The co-expression of CDK4, MDM2 and β-catenin was significantly higher in HGC. A possible interpretation for this result is the accumulation of mutations in lesions of high histological grade, reflecting a genomic complexity in this group of tumors.

In conclusion, the expression of IMP3, CDK4, MDM2 and β-catenin in ENC of short bones phenotypically characterizes these tumors. Their expression has not proven to be useful either as diagnostic markers of these neoplasms or in distinguishing between Enchondroma and Chondrosarcoma, Grade 1. The positivity of IMP3, CDK4, MDM2 and β-catenin antibodies was directly proportional to the increase of histological grade in Chondrosarcoma. The significant immunoexpression of IMP3, CDK4 and MDM2 in metastatic Chondrosarcoma and the lower survival in those with positivity for MDM2, suggest a possible association of these proteins with tumor aggressiveness.

## Funding

The present work was carried out with the support of The São Paulo Research Foundation (FAPESP, 2019/0402–8) and The Coordination for the Improvement of Higher Education Personnel – Brazil (CAPES, financing code 001).

## Declaration of competing interest

The authors declare no conflicts of interest.
